# Final Colour of Ultratranslucent Multilayered Zirconia Veneers, Effect of Thickness, and Resin Cement Shade

**DOI:** 10.1155/2022/2555797

**Published:** 2022-06-01

**Authors:** Seyed Reza Khosravani, Mehdi Abed Kahnamoui, Soodabeh Kimyai, Elmira Jafari Navimipour, Farzaneh Sadeghi Mahounak, Fatemeh Pournaghi Azar

**Affiliations:** ^1^Department of Restorative Dentistry, Faculty of Dentistry, Shahid Beheshti University of Medical Science, Tehran, Iran; ^2^Department of Operative Dentistry, Faculty of Dentistry, Tabriz University of Medical Sciences, Tabriz, Iran; ^3^Dental and Periodontal Research Center, Department of Operative Dentistry, Faculty of Dentistry, Tabriz University of Medical Sciences, Tabriz, Iran

## Abstract

**Background:**

Aesthetic restorations should be able to mimic the natural colour depth of teeth, affected by several factors including material properties. There is a lack of information regarding the effect of cement shade and material thickness on the final colour of ultratranslucent multilayered zirconia veneers.

**Objectives:**

This study evaluated the effect of ceramic thickness and resin cement shade on the final colour of different layers of ultratranslucent multilayered (UTML) zirconia veneers.

**Methods:**

This in vitro study produced 90 rectangular-shaped specimens with nonsintered Katana UTML monolithic zirconia (Kuraray Noritake Dental, Tokyo, Japan), shade A1 blocks. Ceramic samples were prepared in two groups of 0.7 mm and 0.5 mm thicknesses, 45 of each (a: 8 × 11 × 0.5 mm; b: 8 × 11 × 0.7 mm). Specimens of each thickness were further divided into 5 groups: universal, clear, brown, white, and opaque (*n* = 9). Each adhesive resin cement (Panavia V5) was applied between the ceramic samples and composite substrate. The colour values were measured using a spectrophotometer in baseline and after resin cement application according to the CIELab system. For all samples, ΔE00 values were obtained. Data were evaluated with SPSS 25 using the three-way ANOVA test (*p* < 0.05).

**Results:**

The factors of cement shade, ceramic thickness, and ceramic layers have statistically significant effect on ΔE00 values (*p* < 0.001). The results showed lower ΔE00 values with thicker ceramic veneers. Tukey test results showed that the opaque and brown shade had a significantly greater ΔE00 values comparing to universal (*p* = 0.004), clear, and white shades (*p* < 0.001).

**Conclusion:**

The colour change was greater in lower ceramic thickness. Different shades of resin cement and layers of UTML zirconia differently affected the final colour.

## 1. Introduction

Different material selection indications exist including direct versus indirect restorative combinations. Guidance is necessary to help dental professionals make the right decisions between available options to replicate morphology, function, and aesthetics [1].

For the patients' satisfaction with aesthetic effects in anterior teeth, restorative materials whose optical characteristics mimic the natural teeth are necessary [2]. Ceramic system evolution has led to the successful fabrication of aesthetic restorations. Adhesively luted 0.5 mm to 1.0 mm-thick ceramic laminate veneers as a conservative alternative to full coverage restorations present higher translucency, and they are bonded to both prepared and unprepared teeth [3].

The increasing application of zirconia was witnessed due to its unique biocompatibility and biochemical properties [4]. The main drawback of conventional 3Y-TZP ceramics is low translucency, so it cannot imitate the optical characteristics of enamel [5]. Originally, 3Y-TZP was intended as an opaque material, and zirconia-based restorations were acquired by porcelain veneering a zirconia core [6]. However, veneer chipping and fracture, associated with residual thermal stresses induced from the production process, are major technical complications [7]. Moreover, monochromatic blocks were used by dental practitioners, especially for posterior restorations; however, optimum aesthetics cannot be met with monochrome aesthetic materials [3].

Recently, to further improve the aesthetic properties of dental restorations, ultratranslucent multilayered zirconia systems have been introduced. Their microstructure and composition were modified to increase translucency. The aim of multilayered zirconia design is to mimic the shade gradient of natural teeth: where the incisal area of a crown is most translucent, increasing in chroma and opacity towards the gingival area. According to their unique properties, different grades of such zirconia are advocated for various indirect dental restorative applications. Thus, since its first introduction to the dental market, this multilayered monolithic zirconia system has drawn tremendous attention from clinicians and researchers [7–9].

This product represents a remarkable improvement in minimally invasive monolithic CAD/CAM restorations. Ultratranslucent multilayered zirconia (UTML) can be utilized for veneer with a minimum thickness of 0.4 mm and needs simple adhesive cementation [10]. Two clinical studies on monolithic cubic zirconia presented perfect results when used for aesthetic restorations [11, 12].

The mechanical and optical properties of 5Y-ZP (Katana UTML) are between those of 3Y-TZP and lithium disilicate, and the bond strength was comparable to those of lithium disilicate. The use of zirconia for full-arch framework would achieve similar mechanical results for implants and peri-implant tissues, in addition to similar vertical fitting [13]. However, the clinical characteristics of 5Y-ZP require being investigated [14].

An aesthetic restoration should fulfil the optimum shade matching with natural teeth to be clinically successful. Although the ceramic systems promote shade and translucency of restorations, a perfect aesthetic outcome remains unpredictable [15].

Ceramic restorations can be aesthetically influenced by several factors such as underlying tooth colour, resin cement, structure and thickness of ceramic material, and preparation design [3, 16]. Previous research presented that background structure colour has an important role in the final colour of ceramic restorations [15, 17]. More translucent ceramics transmit and scatter more light which leads substrate tooth to have a significant effect over the final shade [15].

Additionally, adhesive cement and ceramic thicknesses have a major influence on the final shade of restoration in respect to the ceramic material and structure. The cement influences approximately ten to fifteen percent of the ultimate optical characteristics of all ceramic restorations [3, 16]. Although some research showed that resin cement type and shade and ceramic thickness affected the final optical outcome of the ceramic veneers, different other studies perhaps will not approve it [4, 18].

Few studies evaluated the zirconia veneers, and there is little information regarding multilayered ultratranslucent zirconia. The final shade of a ceramic restoration made from monochromatic blocks would be similar for each layer after adhesive cementation due to its homogeneous structure, but multilayered blocks have variable colours and translucencies in each layer. Considering the gap of information regarding the possible effect of each layer on the final optical colour of ceramic restoration after the adhesive cementation in multilayered ultratranslucent zirconia, this in vitro study was aimed at investigating the effect of various cement shades and ceramic thickness on the final colour of CAD/CAM ultratranslucent multilayered zirconia ceramic veneers in different layers. The null hypothesis was that the ceramic thickness and cement shade would have no significant effect on the final colour of each layer of a CAD/CAM multilayered ultratranslucent zirconia ceramic.

## 2. Materials and Methods

### 2.1. Study Design

#### 2.1.1. Preparation of Zirconia Samples

**Figure 1:**
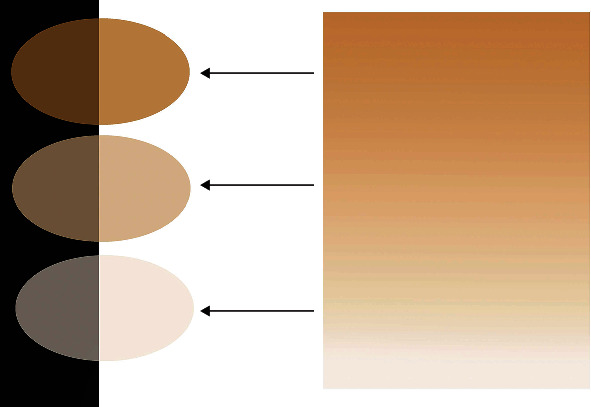
Schematic illustration of the multilayered ceramic sample.

In this in vitro study, 90 specimens were produced with ultratranslucent multilayered monolithic nonsintered zirconia, shade A1 blocks (KATANATM, Kuraray Noritake Dental Inc, Tokyo, Japan). Rectangular-shaped ceramic samples were prepared in two thicknesses (0.7 mm and 0.5 mm), 45 of each group (a: 8 × 11 × 0.5 mm; b: 8 × 11 × 0.7 mm) utilizing CAD/CAM milling (Pixdent, Bonyan Mechatron, Iran). Sintering was carried out in accordance with the manufacturer's instruction, and the surface of specimens was smoothed and polished with 1200-grit silicon carbide abrasive paper [19] (Figure 1).

#### 2.1.2. Fabrication of Substrate

Ninety composite resin specimens (A3 shade, Natural Shade, NOVA DFL, Brazil) were fabricated using rubber mold (8 × 11 × 5 mm) to make the background uniform [20].

### 2.2. Zirconia Sample Cementation

**Figure 2:**
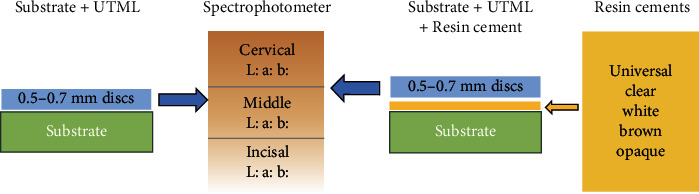
Experimental method flow chart.

Before cementation, airborne particle abrasion protocol (50 mm alumina particles for 10 seconds at 0.2 MPa) was performed. After abrasion, ceramic surface was treated by a single bottle MDP-based adhesive primer (Clearfil Ceramic Primer plus; Kuraray Noritake Dental, Tokyo, Japan). To simulate the clinical scenario for cementation, tooth primer was applied to the composite resin substrate surface (Tooth Primer, Kuraray Noritake Dental, Tokyo, Japan) in accordance with manufacturer's instruction [21]. 45 specimens of each thickness were further divided into 5 groups (*n* = 9), and dual cure adhesive resin cement (Panavia V5, Kuraray Noritake, Tokyo, Japan) in five shades of clear, universal, white, brown, and opaque was applied between the treated surface of ceramic specimen and composite resin substrate. Compressive pressure of 250 gr was applied for 10 s in order to obtain similar thickness of resin cement using universal test machine (Hounsfield 5K, England) 2. The cement was irradiated with a light polymerization device (LITEX 680A Curing Light, Dentamerica, USA) for 40 s in each layer of incisal, body, and cervical (Figure 2).

### 2.3. Colour Measurement

To measure the baseline colour values, ceramic specimens were placed on a white background [22]. The colour coordinate measurements were performed according to the CIELab system utilizing a spectrophotometer (SpectroShade Micro, MHT, Italy).

After cementation process, colour measurement was performed for each cemented specimen in the same manner.

One trained operator performed all spectrophotometer assessments. The instrument was calibrated in accordance with the manufacturer's instructions before and after each measurement. Measurements were made at 3 different points of the cervical, middle, and incisal layers using “tooth area” mode simultaneously, and the mean value was obtained for each portion both in baseline and after cementation. The difference in colour was calculated for each specimen using the ΔE00 (CIE DE2000) formula as follow:

ΔE00 = √((ΔL/KLSL)2 + (ΔC/KcSc)2 + (ΔH/KHSH)2 + Rt(Δc/KcSc) (Δ*Η*/*Κ*HSH)), 

ΔL∗ = L0 (baseline)–L1 (resin cemented), 

Δa∗ = a0 (baseline)–a1 (resin cemented), 

Δb∗ = b0 (baseline)–b1 (resin cemented).

In this study, the threshold of ΔE00 < 2.25 was set as clinically acceptable colour change [23].

### 2.4. Statistical Analysis

Data were presented as means ± SD. To analyse the effect of adhesive resin cement shade and zirconia ceramic thickness on ΔE00 values of different layers, three-way ANOVA and Tukey post hoc test was used. Kolmogorov-Smirnov test was used to assess the normality of ΔE00 values. All statistical analyses were conducted using SPSS for Windows 25.0 (SPSS). Statistical significance was preset at *p* = 0.05.

## 3. Results and Discussion

### 3.1. Results

**Figure 3:**
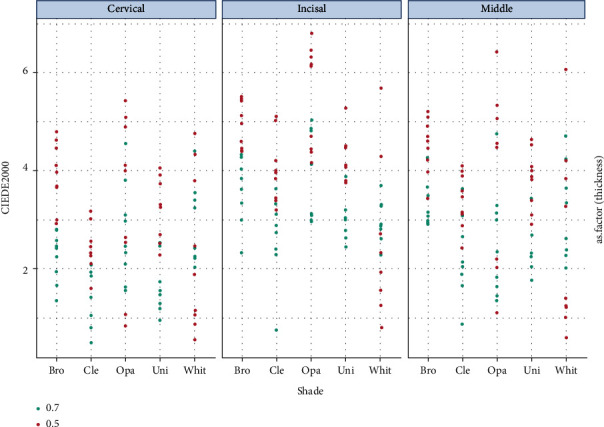
Dotplot of ΔE00 values for different cement shades, layers, and ceramic thicknesses.

Mean ± SD of ΔE00 (CIE DE2000) values for all study groups are presented in Table 1. In both 0.5 and 0.7 mm thick ceramics, brown shade indicated the greatest ΔE00 values (4.49 ± 0.72 and 3.07 ± 0.81, respectively). White and clear shades exhibited the least ΔE00 values (0.5 mm: 2.44 ± 1.62, 0.7 mm: 2.22 ± 0.94, respectively) (Figure 3).

Based on the results of three-way ANOVA, ΔE00 values were significantly influenced by resin cement shade, ceramic thickness, and different layers (*p* < 0.001). Moreover, ceramic thickness and cement shade interaction was statistically significant (*p* < 0.001).

For all shades (except white shade), ΔE00 values of 0.7 mm thickness were lesser than those of 0.5 mm thickness (*p* < 0.001). According to the Tukey test, brown and opaque shades had significantly greater ΔE00 values comparing to universal (*p* = 0.004 and *p* < 0.001, respectively), clear, and white shades (*p* < 0.001).

The obtained results represented a significant difference between ΔE00 values of different layers of ceramic (incisal, body, and cervical) (*p* < 0.001). ΔE00 values were higher for incisal (3.74 ± 1.24) comparing to the other two layers, followed by the body layer (3.18 ± 1.21), with the lowest values for the cervical layer (2.65 ± 1.15) regardless of specimen's thicknesses.

### 3.2. Discussion

Achieving the optimum shade using indirect restorations is important to obtain a perfect outcome in aesthetic restorative treatments, which makes the dentist and the patient satisfied [19].

The optical characteristics of a ceramic restoration are determined by the combination of underlying structure, colour, ceramic system, ceramic thickness, and cement shade [3]. A digital spectrophotometer was used to measure the coordinates and colour difference. While, it is acceptable or perceptible, calculating the colour difference between two samples is very important clinically [24]. CIE DE2000 (ΔE00) colour difference formula was used which improves the correlation between visual judgments (perceptibility) and instrumental colour difference values [25]. In this study, the threshold of ΔE00 < 2.25 was considered as clinically acceptable colour difference [23].

This study's results showed that cement shade, ceramic thickness, and layer had significant effect on the final shade of laminate veneer restorations, so the null hypothesis of study was rejected.

Several ceramic systems are used for ceramic laminates [4]. The most recent measure to enhance the translucency properties of zirconia is to stabilize it with a considerable phase of cubic crystalline which is interspersed with the tetragonal phase [11]. Katana ultratranslucent multilayered (UTML) monolithic zirconia was used in this study since the multilayer approach shows a significant advantage comparing with the one-layer material 10. This prosthetic material intended to replicate the naturality of teeth according to each anatomical region, keeping the highest translucency at the incisal edge [7]. UTML can be utilized for veneer with a minimum thickness of 0.4 mm. In this study, 0.5 and 0.7 mm thick rectangular ceramic samples were selected.

The results showed that the thickness of samples significantly affected the ΔE00 values, and that the values of 0.7 mm thick samples were less than those of 0.5 mm thick samples.

The translucency of lithium disilicate is slightly more than that of 5Y-ZP containing zirconia [26]. As a reference, the translucency parameter is 18.7 for enamel and 16.4 for dentin. Katana UTML has the TP of 11.28.14 shade, and other optical characteristics such as absorption and scattering of light are influenced by the composition and microstructure of the ceramic. Higher yttria content in UTML causes higher cubic content and larger grain size. A larger grain size can effectively reduce the number of grain boundaries in the materials and reduces the grain boundary light scattering. Since c-ZrO2 is optically isotropic, UTML zirconia shows more translucencies, and the ceramic thickness is the main factor to obtain perfect colour matching especially in low thickness [7]. Various studies indicated that the translucency increases by decreasing the ceramic thickness [27, 28]. Moreover, Vichi et al. showed that increased thickness diminishes the diffused reflection effects of the underlay tooth structure, and most of diffused reflection takes place within the ceramic [27]. Increasing ceramic thickness enhances its opacity and prevents light transmission which causes more light scattering and reduction of translucency [26]. Shamseddine et al. showed that 0.4 mm thickness UTML zirconia has the highest translucency, whereas 0.8 and 1 mm thick specimens are similar 7. Increasing the thickness of UTML beyond 0.6 mm does not result in further decrease of translucency [29]. In our study, 0.7 mm thick samples showed less ΔE00 values but were still clinically unacceptable. Similarly, high translucent zirconia with 1-2 mm thickness has a clinically incompatible colour change with opaque cement shade [30]. Çemlekoğlu et al. showed that up to 0.7 mm of these ceramics could not mask the discolorations and suggested that for masking dark substrates, it is better to use low translucent monochromatic ceramics with increased thickness [3].

Adhesive resin cements are commonly utilized to lute ceramic restorations to obtain better esthetical and mechanical properties [25]. Various resin cements are used to lute the ceramic systems. Panavia V5 resin cement was used in this study. It is dual-cured and amine-free, which makes it suitable for cementing even thin veneers without yellowing effect.

Based on this study's results, brown shade showed the highest ΔE00 values in both ceramic thicknesses, followed by opaque shade, which was more than the clinically acceptable threshold (>2.25).

Barath et al. revealed that the shade of luting agent has a considerable effect on the final colour of translucent all-ceramic restorations [31]. Since UTML zirconia is a translucent ceramic, the colour of resin cement is mainly reflected via restoration. It is important for the clinicians to predict the translucency of ceramic laminates when the resin cement is placed under them rather than relying solely on the original translucency of material. By brown shade, cement *L* values decreased, and “*a*” increased. Although it is a translucent cement (11.2%), increasing “a” coordinate due to pigmentation lowers its translucency (L) and high ΔE00 values are observed. Chen et al. also demonstrated a significant increase in “*a*” value in brown colour [32]. Universal and clear shades have the lowest ΔE00 values, although clinically unacceptable (>2.25). Both two cements are translucent, especially clear shade, and may have the least effect on the final colour. Xing et al. showed that translucent shades slightly increased the brightness and decreased the chroma of ceramics which had no significant effect on the final colour [24].

The results showed that ΔE00 values of opaque cement were significantly greater. Niu et al. and Chang et al. revealed that the opaque shade remarkably enhances the *L*∗ value and brightness and causes less chroma [33, 34]. Commonly, masking the undesirable shade of background structure is the clinical purpose for opaque shade application, which alters the values [25].

Different studies showed that using opaque cements improves the final colour of restorations when the underlying structure shade is darker [17, 35, 36]. Dede et al. showed that just C2 shade presented the improved final colour values when using opaque shade cements [25]. Furthermore, Barath et al. showed that translucent luting agents have less effect when used on a darker background, and opaque resin cements indicates better results with dark background along with dark ceramics [31].

Bayindir et al. showed that clear and opaque shades decrease the translucency parameter of translucent zirconia after cementation. However, TP values of the specimens of all thicknesses cemented with transparent shade were higher than those of cemented with opaque shade [30].

Based on the results, the interaction of cement shade and ceramic thickness was significant. Xing et al. revealed that the effect of luting agent shade on the final colour of laminate veneers was associated with ceramic thickness [24].

This study's results showed that various layers of multilayer ceramic significantly affected the ΔE00. The incisal layer showed the highest ΔE00 values due to lower colour pigment ingredients in this area compared to the body and cervical layers [3].

UTML zirconia used in the present study has 43% of light transmittance across layers. Four layers of DEL, FTL, STL, and ENL are presented in this zirconia ceramic. Harada et al. indicated that UTML has higher transmittance (Tt%) when compared with other types of zirconia [8]. Moreover, they showed that transmittance values of each layer of ultratranslucent multilayered zirconia are different 38. Ueda et al. reported that *L* increased from DEL to ENL, while “*a*” and “*b*” values diminished across the same layer sequence [37]. Pigmentation makes a change toward red and yellow, which makes ENL more transparent than DEL. Hence, cervical zone that includes the dentin layer has less translucency, in which the ΔE00 values are less than the incisal zone, which is the ENL of laminate veneer. On the contrary, Shamseddine and Majzoub showed that relative translucency values (TPS) were similar across all four layers for all different thicknesses. They suggested that four-layered UTML material, which should provide a more natural appearance of monolithic restorations, may not present visually perceived differences in translucency between layers [29]. Kolakarnprasert et al. showed that multilayer zirconia had Fe additives in its dentin layer, whereas the enamel layer did not, which affects both TP values and LTD (low temperature degradation) behavior [7].

As previously investigated, different ceramic systems show different optical properties. Masking properties of different ceramics for veneer restorations depends on the translucency and thickness of material [38, 39]. The translucency in UTML zirconia remains a challenge because the presence of crystals and pigments attenuates the light and the composition, and the microstructure of each layer material may influence translucency due to difference between the refractive index of crystals and size of filler particles [21]. Determining the role of optical characteristics of multilayered zirconia in the aesthetic appearance of ceramic restorations needs more clinical investigations especially for thin laminates.

One of the limitations of this study was that one shade of background composite was used. As it was mentioned, opaque resin cements can improve the colour in darker tooth shades and the underlying tooth structure may affect the final colour of restoration; therefore, further studies are needed to evaluate the effects of a wider range of underlying shades on the colour of ultratranslucent monolithic zirconia restorations. Since the samples tested in this study were rectangular-shaped rather than ceramic veneer restorations, the clinical conditions are not completely simulated, and it is suggested to conduct a clinical study to completely assess the final shade of ceramic veneers.

## 4. Conclusions

In short, considering the limits of this investigation, the following conclusions were drawn: Firstly, the colour change was greater in lower ceramic thickness. Secondly, various shades of cement considerably affect the final colour of ceramic veneers. Finally, the layers of UTML zirconia differently affected the final colour.

## Figures and Tables

**Table 1:** Mean ± standard deviationΔE00 values for the study groups.

	Universal		Clear		Brown		White		Opaque	
	0.5 mm	0.7 mm	0.5 mm	0.7 mm	0.5 mm	0.7 mm	0.5 mm	0.7 mm	0.5 mm	0.7 mm
Cervical	3.24 ± 0.62	1.75 ± 0.60	2.45 ± 0.46	1.62 ± 0.76	3.91 ± 0.66	2.25 ± 0.50	2.32 ± 1.59	2.82 ± 0.85	3.40 ± 1.71	2.72 ± 0.99
Body	3.82 ± 0.59	2.59 ± 0.59	3.40 ± 0.54	2.24 ± 0.81	4.51 ± 0.56	3.27 ± 0.45	2.55 ± 1.87	3.17 ± 0.91	3.69 ± 1.86	2.54 ± 1.11
Incisal	4.17 ± 0.51	3.08 ± 0.57	4.01 ± 0.67	2.79 ± 0.93	5.04 ± 0.45	3.69 ± 0.69	2.46 ± 1.57	2.86 ± 0.54	5.50 ± 1.04	3.78 ± 0.91
Total	3.74 ± 0.67	2.47 ± 0.79	3.29 ± 0.85	2.22 ± 0.94	4.49 ± 0.72	3.07 ± 0.81	2.44 ± 1.62	2.95 ± 0.77	4.20 ± 1.78	3.01 ± 1.11

## Data Availability

The data used to support the findings of this study are available from the corresponding author, Dr. Fatemeh Pournaghi Azar, upon request (email address: pournaghiazarf@gmail.com).
